# Tobacco Xenobiotics Release Nitric Oxide

**DOI:** 10.1186/1617-9625-1-19

**Published:** 2003-09-15

**Authors:** EWN Lam, EE Kelley, SM Martin, GR Buettner

**Affiliations:** 1 Department of Dentistry, Faculty of Medicine and Dentistry, University of Alberta, Edmonton, Canada; 2 Free Radical & Radiation Biology Graduate Program and Electron Spin Resonance Facility, The University of Iowa College of Medicine, Iowa City, Iowa, USA

## Abstract

Many xenobiotic compounds exert their actions through the release of free radicals and related oxidants [1,2], bringing about unwanted biological effects [3]. Indeed, oxidative events may play a significant role in tobacco toxicity from cigarette smoke. Here, we demonstrate the direct in vitro release of the free radical nitric oxide (^•^NO) from extracts and components of smokeless tobacco, including nicotine, nitrosonornicotine (NNN) and 4-(methyl-N-nitrosamino)-1-(3-pyridyl)-1-butanone (NNK) in phosphate buffered saline and human saliva using electron spin resonance and chemiluminescence detection. Our findings suggest that tobacco xenobiotics represent as yet unrecognized sources of ^•^NO in the body.

## Introduction

Whether generated intracellularly, or exogenously delivered, the diatomic free radical nitric oxide (^•^NO) is rapidly disseminated throughout the body, affecting key biological processes. Supra-physiologic ^•^NO concentrations favor the formation of a potent biological oxidant; peroxynitrite (ONOO^-^), the reaction product of ^•^NO and the oxygen-centered free radical, superoxide, O_2_^•- ^[4]. Numerous cytotoxic lesions have been attributed to ONOO^-^, including lipid peroxidation, protein thiol oxidation, inhibition of Fe-S enzyme systems, and oxidative DNA lesions such as strand breaks and base modifications, to name some [4-6].

Of the over 30 carcinogens found in tobacco, the nitrosamine compounds, nitrosonornicotine (NNN) and 4-(methylnitrosamino)-1-(3-pyridyl)-1-butanone (NNK) are thought to be the major contributors to the carcinogenic activity of nicotine and tobacco [7,8]. NNN and NNK are formed during the curing, aging, and fermentation of tobacco, as well as during nicotine metabolism. Already, ^•^NO generation has been demonstrated in cigarette smoke [9]. The structural similarities between NNN and NNK, and other known therapeutic and experimental ^•^NO-releasing compounds suggest that these nitrosamines may be novel ^• ^NO-releasing agents in tobacco [10,11]. Indeed, NNK has been shown to generate DNA strand breaks, as well as induce the formation of DNA adducts, including methylated DNA [12,13].

Here, we demonstrate, using both direct and indirect methods, the *in vitro *release of ^•^NO from extracts and components of smokeless tobacco, including nicotine, and the nitrosamine metabolites of tobacco, nitrosonornicotine (NNN) and 4-(methyl-N-nitrosamino)-1-(3-pyridyl)-1-butanone (NNK).

## Materials and methods

### Tobacco xenobiotic preparations

Experiments were conducted in phosphate-buffered saline (PBS) at pH 7.4 or unstimulated human saliva obtained from healthy, non-users of tobacco, without clinical evidence of periodontal disease. We estimated the mass of a "pinch" of smokeless tobacco to be approximately 2.2 g, and suspended this (Copenhagen^® ^brand, National Tobacco Co., Ltd., Pointe Claire, QB) in 4.4 mL of PBS or saliva. The amount of nicotine in this preparation has been determined previously to be 12 ± 0.7 mg per g tobacco [8]. Therefore, 26.4 mg of nicotine (Sigma Chemical Co., St Louis, MO) was used for the assays. Ten mg of NNN and NNK (Midwest Research Institute, St. Louis, MO) was used for ^•^NO determinations. Each of these prepared solutions was purged with argon gas, and incubated at 37°C for 20 min in an air-tight container before being assayed for ^•^NO.

### EPR spin trapping

Each xenobiotic preparation was incubated with a 10 mM solution of the iron (II)/N-methyl-D-glucamine dithiocarbamate, Fe^2+^(MGD)_2_, spin trap at 37°C for 20 min so that the final concentration of the spin trap was 1 mM [14]. Each 500 μL solution was then quickly transferred to an argon-purged flat cell, and EPR spectra were collected with a Bruker (Billerica, MA, USA) X-band EMX spectrometer operating at 9.75 GHz, receiver gain of 2 × 10^4^, modulation amplitude of 1 G, sweep time of 83 s, and a field center of 3418 G for ^•^NO-Fe^2+^(MGD)_2_. Each spectrum represents the signal-averaged sum of 15 acquisitions.

### Chemiluminescent detection

Fifty μL of each xenobiotic solution was injected into a Sievers 280 Nitric Oxide Analyzer (Boulder, CO, USA) containing a reducing agent, KI, potassium iodide (5.9 mM) in glacial acetic acid [14]. Standardization was accomplished by injecting various concentrations of a standard solution of NaNO_2 _into the same reducing environment. Samples were run in triplicate.

## Results and Discussion

**Figure 1:**
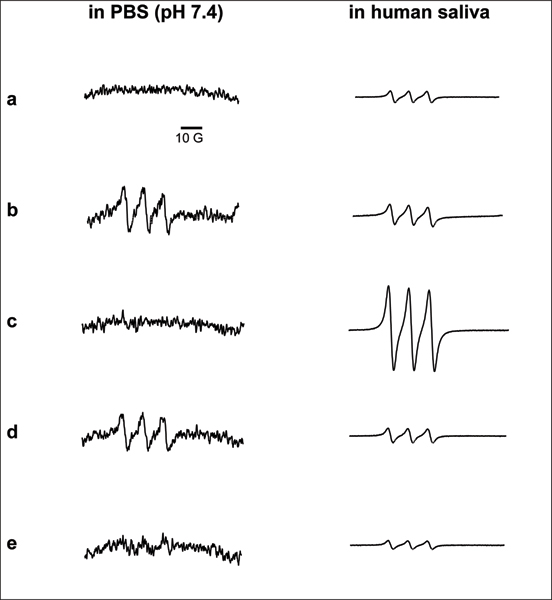
**EPR spectra of the ^•^NO-Fe(MGD)_2 _spin adduct formed from the release of ^•^NO from tobacco xenobiotics in phosphate buffered-saline at pH 7.4 (left column) and human saliva (right column)**. The hyperfine splitting value, a_N_, of these spectra is 12.4 G and the isotropic nuclear g value, g_iso_, is 2.04. **(a) **500 μL phosphate buffered saline (left column) or whole human saliva (right column); **(b) **500 μL of a 1:1 w/v extract of smokeless tobacco; **(c) **3.48 M (26.7 μG) nicotine; **(d) **1.1 M (10 mg) nitrosonornicotine (NNN); and **(e) **1.0 M (10 mg) 4-(methyl-N-nitrosamino)-1-(3-pyridyl)-1-butanone (NNK). Each spectrum represents 15 summed signal acquisitions acquired using a receiver gain of 2 × 10^4 ^and modulation amplitude of 1 G. The ordinate scale is ± 1 × 10^3 ^arbitrary units for all spectra in whole human saliva.

Electron paramagnetic resonance (EPR) spin trapping was used to identify ^•^NO release from tobacco xenobiotics. The EPR-silent ^•^NO spin trap iron (II)/N-methyl-D-glucamine dithiocarbamate, Fe^2+^(MGD)_2_, coordinates the free ^•^NO radical in aqueous solution, forming a stable, EPR-visible spin adduct, ^•^NO-Fe^2+^(MGD)_2_. This species yields a characteristic three-line EPR spectrum with an inter-peak hyperfine splitting value, a_N_, of 12.4 G and an isotropic nuclear g value, g_iso_, of 2.04, both of which are characteristic of trapped ^•^NO [14] (Figure 1). We observed unstimulated ^•^NO release from smokeless tobacco extract and NNN, and weak release from NNK in PBS. Free ^•^NO was not detected from pure nicotine under these conditions. However, given its chemical structure, we would not expect to observe an EPR signal from nicotine. When these experiments were performed in human saliva under identical conditions, we observed substantially stronger EPR signals. We believe this increased signal strength to be derived, in part, from the reduction of salivary NO_2_^- ^by cytochrome cd_1 _nitrite reductase found in some salivary bacteria [15,16]. Under these conditions, we observed a substantial EPR signal from nicotine in human saliva. The intensity of this signal suggests there to be substantial biotransformation of nicotine, facilitating the release of ^•^NO. These results suggest that nicotine-derived ^•^NO may substantially contribute to the systemic ^•^NO load to an extent not previously recognized.

As EPR is only semi-quantifiable, we used a chemiluminescence technique to determine ^•^NO concentrations derived from smokeless tobacco xenobiotics. This technique, however, detects only the end-product of ^•^NO oxidation, namely NO_2_^-^. The use of this technique together with EPR spin trapping is considered complementary [14]. The results of these experiments are summarized in Table 1. Briefly, phosphate buffered saline and human saliva generate 5 ± 1 μM and 38 ± 17 μM ^•^NO, respectively, while extracts of smokeless tobacco in these fluids produced 1100 ± 50 μM ^•^NO (2.53 ± 0.10 mmol ^•^NO/g ST) and 1380 ± 80 μM (2.76 ± 0.16 mmol ^•^NO/g ST), respectively. The similarity of these results may reflect the high inherent NO_2_^- ^content of processed smokeless tobacco [5]. We were unable to detect ^•^NO from nicotine, NNN and NNK in PBS; the concentrations of NNN- and NNK-derived ^•^NO were likely below the detection threshold of the technique (0.2 μM). When nicotine, NNN and NNK were incubated in human saliva, we detected micromolar (or nanomole quantities per milligram xenobiotic) of ^•^NO: 150 μM ± 12 from nicotine (2.81 ± 0.23 nmol/mg nicotine), 121 ± 6 μM from NNN (5.90 ± 0.30 nmol/mg NNN) and 113 ± 5 μM from NNK (5.45 ± 0.25 nmol/mg NNK), respectively. As nicotine and the nitrosamine metabolites are found in milligram and microgram quantities per gram of smokeless tobacco, the putative ^•^NO load derived from these compounds is substantial. Moreover, the importance of saliva in ^•^NO release from these compounds is notable.

**Table 1:** Chemiluminescent detection of ^•^NO

	**Total ^•^NO observed**	
	
	**in PBS (pH 7.4)**	**in human saliva**
**PBS**	5 ± 1 μM^a^	-

**Whole human saliva (WHS)**	-	38 ± 17 μM^a^

**Smokeless tobacco (ST)**	1100 ± 50 μM^a^ 2.53 ± 0.10 μmol/g ST	1380 ± 80 μM^a^ 2.76 ± 0.16 μmol/g ST

**Nicotine**	< 0.02 μM^b^	150 ± 12 μM^a^ 2.81 ± 0.23 nmol/mg nicotine

**Nitrosonornicotine (NNN)**	< 0.02 μM^b^	121 ± 6 μM^a^ .90 ± 0.30 nmol/mg NNN

**4-(Methyl-N-nitrosamino)-1-(3- pyridyl)-1-butanone (NNK)**	< 0.02 μM^b^	113 ± 5 μM^a^ 5.45 ± 0.25 nmol/mg NNK

^a^This is the apparent concentration of ^•^NO present as NO_2_^- ^in the aqueous incubation injected into the Sievers NOA analyzer.

^b^The limit of detection with our experimental conditions.

Although others have reported free radical, and in particular, O_2_^•- ^production in cells exposed to smokeless tobacco and nicotine [17-19], none identified free radical release directly from smokeless tobacco xenobiotics. Tobacco xenobiotics represent as yet unrecognized sources of ^•^NO in the body. Indeed tobacco-derived ^•^NO may have widespread biological implications for tobacco users. Our results also lead us to speculate that ^•^NO and nitrosative events may play a role in tobacco toxicity in the oral cavity and aerodigestive tract.

## Competing interests

The authors declare that they have no competing interests.
